# Efficacy and Safety of Switching from Tafluprost to a Tafluprost/Timolol Fixed Combination in Patients With Primary Open-Angle Glaucoma

**DOI:** 10.2174/1874364101812010121

**Published:** 2018-06-29

**Authors:** Kenji Inoue, Takeaki Ueda, Kyoko Ishida, Goji Tomita

**Affiliations:** 1 Inouye Eye Hospital, Tokyo, Japan; 2 Department of Ophthalmology, Toho University Ohashi Medical Center, Tokyo, Japan

**Keywords:** Tafluprost, Tafluprost/timolol fixed combination, Primary open-angle glaucoma, Intraocular pressure, Safety, Efficacy

## Abstract

**Background:**

The Tafluprost/Timolol Fixed Combination (TTFC) has demonstrated efficacy and safety in reducing Intraocular Pressure (IOP). However, direct comparisons of switching from tafluprost to TTFC are limited.

**Objective:**

To investigate the efficacy and safety of switching from tafluprost to TTFC in patients with Primary Open-Angle Glaucoma (POAG).

**Methods:**

Thirty-four eyes (34 patients) with POAG that did not achieve adequate IOP reduction on tafluprost were switched to TTFC with no washout period. IOP, systolic/diastolic blood pressure and pulse rate were measured 1 and 3 months later and compared with baseline values. All participants were asked about specific adverse reactions after 1 and 3 months of treatment. Patients also completed a questionnaire about preference and adherence after 1 month of treatment.

**Results:**

Mean IOP after 1 and 3 months was significantly lower than at baseline (14.2 ± 2.1 mmHg and 14.1 ± 2.3 mmHg, respectively, vs 16.0 ± 2.0 mmHg, P < 0.0001). Systolic/diastolic blood pressure and pulse rate were not significantly different from baseline after 1 and 3 months. The questionnaire indicated that the frequency of missing a dose was not different before (27.3%) or after (18.2%) switching to TTFC (P = 0.2371). There were five reports of adverse reactions (14.7%), including a corneal epithelium disorder, ocular irritation, skin irritation at the wrist, and chest pain. Two patients (5.9%) withdrew because of adverse reactions.

**Conclusion:**

Switching from tafluprost to TTFC achieved IOP control safely and was well accepted by patients.

## 1 INTRODUCTION

Glaucoma is often initially treated with a single medication that lowers Intraocular Pressure (IOP). However, when a single medication has inadequate IOP-lowering efficacy, it is necessary to change the therapy or add other eye drops [1]. Multiple medications increase the number of eye drops that need to be instilled, which can lead to poor patient adherence [2]. Therefore, a fixed-combination eye drop that contains two medications is recommended.

Prostaglandin (PG) analogs are generally the first choice for treatment of glaucoma because they are effective in reducing IOP, have few systemic adverse effects, and allow a convenient once-daily administration protocol [3]. A switch from a PG analog to a PG/timolol fixed combination eye drop is common when more aggressive therapy is required [4] because of the high patient compliance and ability to continue on a once-daily administration protocol.

The preservative-containing Tafluprost/Timolol Fixed Combination (TTFC) was approved for use in Japan in 2014 and the preservative-free agent is used in many other parts of the world. TTFC has demonstrated efficacy and safety in reducing IOP in some studies [5-11]. However, with the exception of some studies in Japan [5, 6, 8] and one study in Germany [7], direct comparisons of switching from tafluprost to TTFC are limited. The efficacy and safety of TTFC have been investigated in patients with all types of glaucoma and Ocular Hypertension (OH) [7, 8], but there are no reports on the efficacy and safety of TTFC in Japanese patients with primary open-angle glaucoma (POAG). Furthermore, the previous studies of TTFC in Japan [5, 6] were conducted as clinical trials, and there have been no direct comparisons of the efficacy and safety of tafluprost and TTFC in patients with POAG and OH in routine clinical practice.

In this prospective study, we investigated the efficacy and safety of IOP reduction and patient adherence in routine clinical practice in Japanese patients with POAG who were switched from tafluprost to TTFC.

## 2 MATERIALS AND METHODS

The study protocol was approved by the ethical committee at our hospital and all participants provided written informed consent before any study procedure or examination was performed. The study conduct adhered to the tenets of the Declaration of Helsinki.

The study was conducted between July 2015 and May 2017 at Inouye Eye Hospital (Tokyo, Japan) and included 34 eyes of 34 patients with POAG who had had an inadequate decrease in IOP after more than 3 months of treatment with tafluprost 0.0015% (Tapros^®^; Santen Pharmaceutical Co. Ltd., Osaka, Japan), necessitating a change in IOP-lowering medication. In cases where both eyes qualified for inclusion in the study, the eye with the higher IOP was selected as the study eye. If both eyes had the same IOP, the right eye was selected as the study eye.

The study participants discontinued using tafluprost once daily at night and switched to TTFC (Tapcom®; Santen Pharmaceutical Co. Ltd.) once daily at night with no washout period in between.

**Fig. (1):**
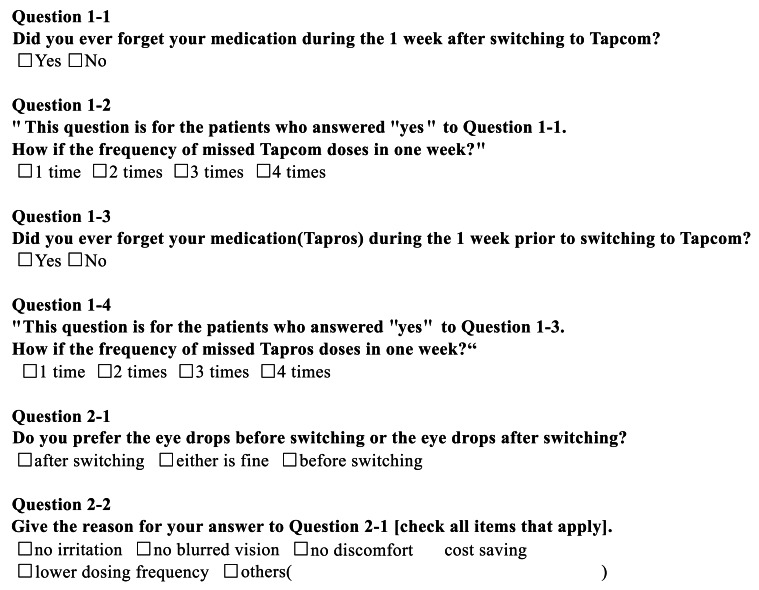
Questionnaire on patient preference, adherence, and specific adverse reactions.

All eyes underwent ophthalmic examination, including IOP measurement (Goldmann tonometry), before and after using the study medication for 1 month and 3 months. Systolic/diastolic Blood Pressure (BP) and pulse rate were measured using an Udex super type pulsometer, Elquest Inc., (Chiba, Japan) at each time point. The width and rate of IOP reduction from baseline values were assessed after 1 and 3 months of treatment. The study participants were asked about specific adverse reactions after 1 and 3 months of treatment. Preference and adherence were investigated using a questionnaire after 1 month of treatment (Fig. **1**).

Analysis of variance and Bonferroni/Dunn analyses were used to compare the IOP, systolic/diastolic BP, and pulse rate values obtained before and after administration of TTFC. The width and rate of IOP reduction from baseline at 1 and 3 months were compared using the Wilcoxon signed-rank test. We calculated that a target sample size [5] of 26 would be required to allow detection of a 2-mmHg difference in IOP after switching to TTFC, with an expected standard deviation of 3.5 mmHg at a power of 0.80. A *p*-value <0.05 was considered statistically significant.

## 3 RESULTS

**Fig. (2):**
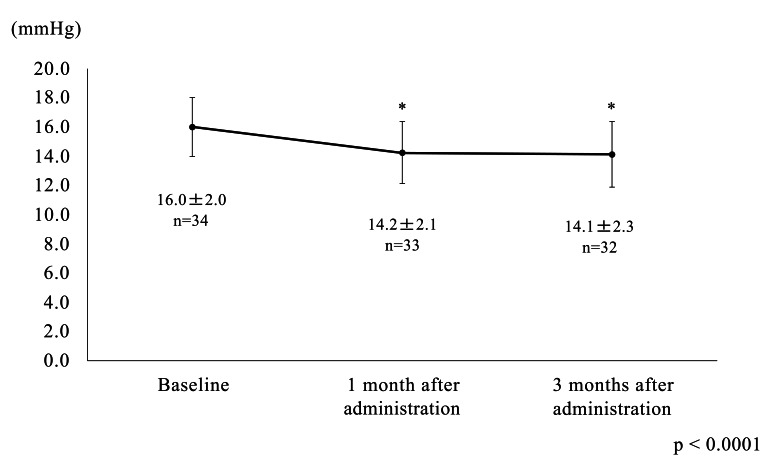
Changes in mean intraocular pressure before and after switching to the fixed combination of tafluprost/timolol. *p < 0.0001.

The patients comprised 14 men and 20 women with a mean age at baseline of 66.6 ± 11.4 (range 27–82) years. The mean deviation on the Humphrey visual field test program 30-2 SITA Standard was -6.63 ± 5.59 (range -19.33 ~ 0.02) dB. Six eyes had POAG and 28 had normal-tension glaucoma. The IOP was significantly lower after 1 (14.2 ± 2.1 mmHg) and 3 (14.1 ± 2.3 mmHg) months of using the study medication when compared with baseline (16.0 ± 2.0 mmHg, both *P* < 0.0001; Fig. **2**). There was no statistically significant difference in width or rate of IOP reduction after 1 and 3 months of treatment with the study medication.

There were also no statistically significant differences in pulse rate or systolic/diastolic BP between baseline and 1 and 3 months after switching medication (pulse rate, *p* = 0.3433; systolic BP, *p* = 0.5367; diastolic BP, *p* = 0.7645, Table **1**).

One patient dropped out of the study because of an adverse effect after 5 days of using the study medication, leaving 33 patients available to complete the questionnaire after 1 month of treatment. Six patients (18.2%) answered ‘yes’ and 27 (81.8%) answered ‘no’ when asked if they had ever forgotten to use their medication during the first week after switching to the study medication. The 6 patients who answered ‘yes’ to the above question reported missing one dose. When asked if they had ever forgotten to use their medication in the week before switching to the trial medication, 9 patients (27.3%) answered ‘yes’ and 22 (66.7%) answered ‘no’ (2 [6.0%] did not answer this question); 7 (77.8%) of the 9 patients who answered ‘yes’ missed one dose, 1 (11.1%) missed two doses, and 1 (11.1%) did not answer this question.

Six patients (18.2%) reported a preference for the trial medication, 21 (63.6%) had no preference, and 6 (18.2%) preferred tafluprost alone. Two of the 6 patients who preferred the trial medication reported that they did so because there was ‘no blurred vision’, 1 because of the ‘lower dosing frequency’, and 1 because of ‘feeling refreshed’. Four of the 6 patients who preferred to use tafluprost alone did so because of ‘no ocular irritation’ and 1 because of ‘no discomfort’.

Five patients (14.7%) experienced adverse reactions, included irritation of the skin at the wrist after 5 days, ocular irritation at 1 month, chest pain at 1 month, a corneal epithelium disorder at 1 and 3 months, and a corneal epithelium disorder at 3 months (all in 1 patient each). Two patients (5.9%) discontinued using the TTFC because of irritation of skin at the wrist (*n* = 1) and chest pain (*n* = 1).

## 4 DISCUSSION

In this study, the width and rate of IOP reduction from baseline values was significant 3 months after the patients switched from tafluprost alone to the TTFC (1.8 ± 1.8 mmHg and 11.2 ± 10.3%, respectively). In a previous study in which timolol was added to tafluprost, the width and rate of IOP reduction were 2.2 ± 1.8 mmHg and 11.4%, respectively [5]. There was no significant difference in the efficacy of IOP reduction between switching from tafluprost to TTFC in our present study and the previous study in which timolol was added to tafluprost [5].

The IOP-lowering effect of a prostaglandin/timolol fixed combination eye drop formulation has been of concern because of the need to decrease the frequency of instillation of timolol from twice daily to once daily. Several studies have reported that penetration of timolol increases with increasing pH, so in the present study we used a pH-adjusted formulation to increase the penetration of timolol to the level that would be achieved by once-daily administration [12]. Our findings are very similar to those of another study in which timolol was added to tafluprost [5].

When switching from tafluprost to a fixed combination of tafluprost/timolol in patients with POAG, decreases in IOP width of 2.6 ± 1.8 mmHg [5] and 1.7 mmHg [6] during 4 weeks of follow-up have been reported, as well as a decrease of 2.2 mmHg during 52 weeks of follow-up [6].There have been reports of IOP reduction rates of 10.0% [6] and 13.5% [5] during 4 weeks of follow-up, and 12.9% during 52 weeks of follow-up [6]. Again, there was no significant difference between our findings and those of the above-mentioned studies [5, 6]. However, the previous studies that investigated in patients with glaucoma and OH reported that the width and rate of IOP reduction were 3.6–5.1 mmHg and 18.0–24.0% during 4–16 weeks of follow-up [7] and 2.5 mmHg and 15.0% during 3 months of follow-up [8].

When switching from latanoprost to a fixed combination of latanoprost/timolol, the width of IOP reduction was 2.4 ± 2.2 mmHg after 3 months and 2.1 ± 2.3 mmHg after 6 months, with respective IOP reduction rates of 13.1 ± 10.9% and 11.2 ± 11.8% [13]. When switching from travoprost to a fixed combination of travoprost/timolol, the respective width and rate of IOP reduction were 1.9 ± 1.7 mmHg and 10.5 ± 9.9% after 1 month and 2.1 ± 1.7 mmHg and 11.9 ± 9.6% after 3 months [14]. The findings in those two studies are not significantly different from those in our present study.

In our study, neither systolic/diastolic BP nor pulse rate values were significantly different after 1 and 3 months of treatment from those at baseline, which is consistent with earlier studies reporting no significant difference in systolic/diastolic BP from baseline after 2 and 4 weeks of treatment [5, 6]. In one of the previous studies, the pulse rate values after 2 and 4 weeks were significantly lower than the pulse rate at baseline [5]; however, in the other study, the difference detected at 2 weeks was no longer present at 4 weeks [6]. The reduction in pulse rate width after 2 and 4 weeks was -2.0 ± 7.5/min and -1.3 ± 7.9/min, respectively, but these values were within normal limits [5]. These reports also suggested that TTFC had no adverse effects on the circulatory system.

Adverse reactions occurred in 5 patients (14.7%) and included a corneal epithelium disorder, ocular irritation, irritation of the skin at the wrist, and chest pain. These adverse reactions were the same as those reported in the previous studies [5-8]. Adverse reactions occurred in 10.5% [5] and 8.3% [6] in the previous studies, which is similar to the frequency in our present study.

A previous study reported a dropout rate of 6.5% (2 cases) because of adverse reactions (itch and bradycardia) when patients were switched from latanoprost to a fixed combination of latanoprost/timolol [13]. In another study, 1 patient (3.3%) dropped out because of blurred vision after switching from travoprost to a fixed combination of travoprost/timolol [14]. In our study, 2 patients (5.9%) dropped out because of irritation of the skin at the wrist and chest pain. The dropout rate in our study is similar to that in the previously reported studies [13, 14]. Therefore, we consider that the safety of TTFC is similar to that of other fixed combinations.

In this study, we investigated patient preference and adherence by questionnaire, which has not been undertaken in previous studies. Adherence to medication did not change after switching to TTFC because the dosing frequency was the same as that for tafluprost used alone. Moreover, more than 80% of patients in our present study had a favorable or unchanged impression of TTFC after switching, suggesting that both fewer adverse reactions and improved adherence can be expected with this medication.

The main limitation of this study is that it only evaluated the short-term safety and efficacy of IOP reduction. Future studies should include larger sample sizes and long-term follow-up to confirm the utility of switching from tafluprost to TTFC in patients with POAG.

## CONCLUSION

In this study, we prospectively investigated the efficacy and safety of switching from tafluprost to a TTFC in Japanese patients with POAG in routine clinical practice. Switching to the TTFC was safe and effective for IOP reduction with no decrease in patient adherence. The TTFC is an acceptable treatment option for patients with POAG.

## Figures and Tables

**Table 1:** Differences in pulse rate or systolic/diastolic BP between baseline and 1 and 3 months after switching medication (pulse rate, *p* = 0.3433; systolic BP, *p* = 0.5367; diastolic BP, *p* = 0.7645).

–	–	Baseline	1 month after administration	3 months after administration	P-value
Blood pressure	Systolic mmHg	134±19	130±23	134±19	0.5367
–	Diastolic mmHg	74±10	73±9	75±10	0.7645
pulse rate (/min)	–	74±10	73±8	75±10	0.3433
